# Decompression alone versus fusion and Coflex in the treatment of lumbar degenerative disease: A network meta-analysis

**DOI:** 10.1097/MD.0000000000019457

**Published:** 2020-03-13

**Authors:** Yunpeng Fan, Liulong Zhu

**Affiliations:** a Department of Orthopedic Surgery, The Affiliated Hangzhou Hospital of Nanjing Medical University; b The Affiliated Hangzhou First People's Hospital, Zhejiang University School of Medicine, Hangzhou, China.

**Keywords:** Coflex, decompression alone, lumbar interbody fusion, network meta-analysis

## Abstract

**Background:**

Lumbar degenerative disease (LDD) is a very common disease. And decompression alone, posterior lumbar interbody fusion (PLIF), and interspinous device (Coflex) are generally accepted surgical techniques. However, the effectiveness and safety of the above techniques are still not clear. Network meta-analysis a comprehensive technique can compare multiple treatments based on indirect dates and all interventions are evaluated and ranked simultaneously. To figure out this problem and offer a better choice for LDD, we performed this network meta-analysis.

**Methods:**

PubMed and WanFang databases were searched based on the following key words, “Coflex,” “decompression,” “PLIF,” “Posterior Lumbar Interbody Fusion,” “Coflex” “Lumbar interbody Fusion.” Then the studies were sorted out on the basis of inclusion criteria and exclusion criteria. A network meta-analysis was performed using The University of Auckland, Auckland city, New Zealand R 3.5.3 software.

**Results:**

A total of 10 eligible literatures were finally screened, including 946 patients. All studies were randomized controlled trials (RCTs). Compared with decompression alone group, there were no significant differences of Oswestry Disability Index (ODI) in Coflex and lumbar interbody fusion groups after surgery. However, Coflex and PLIF were better in decreasing Visual Analogue Scale (VAS) score compared with decompression alone. Furthermore, we found Coflex have a less complication incidence rate.

**Conclusion:**

Compared with decompression alone, Coflex and lumbar interbody fusion had the similar effectiveness in improving lumbar function and quality of life. However, the latter 2 techniques were better in relieving pain. Furthermore, Coflex included a lower complication incidence rate. So we suggested that Coflex technique was a better choice to cue lumbar spinal stenosis (LSS).

LEVEL OF EVIDENCE: Systematic review and meta-analysis, level I.

## 1 Introduction

Lumbar degenerative disease (LDD) is a degenerative disease and common happened in the elderly. The number of patients suffering low-back and leg pain was up to 12% world population and will be double by 2050.^[1–3]^ LDD mainly restrict the lumbar function, walking ability and quality of life. Most of lumbar spinal stenosis can be treated by conservative methods. However, some patients are still pain after conservative treatment. So the surgery will be a better choice as to these patients.^[4,5]^

Recently, the superiority of Coflex over decompression alone and posterior lumbar interbody fusion (PLIF) to treat LDD generated a heated controversy.^[6–8]^ Decompression alone like percutaneous transforaminal endoscopic discectomy (PTED) cues the LDD with posterior column lumbar structures preserved is advocated as a mean to relieve pain and decrease blood loss.^[9]^ Although PTED avoids disruption of musculature and ligaments structures, PTED technique demands a longer learning curve and lacks of fixation to improve lumbar stability. Coflex and lumbar interbody fusion include decompression and device fixation. Their advantages include an easier surgery procedure and the “open” visualization to avoid inadequate decompression.^[10,11]^ However, both later 2 surgeries will cost more money and prolong the hospital stays. The acceptability is lower to patients especially to elderly than PTED.^[12,13]^

Network meta-analysis a more comprehensive technique compared with the meta-analysis has been developed. It can compare multiple treatments based on indirect dates and all interventions are evaluated and ranked simultaneously.^[14–16]^ Therefore, we performed a network meta-analysis to compare effectiveness and safety of decompression alone, Coflex and lumbar interbody fusion surgeries to provide a better surgical options for LDD treatments.

## 2 Methods

### 2.1 Literature search

**Figure 1:**
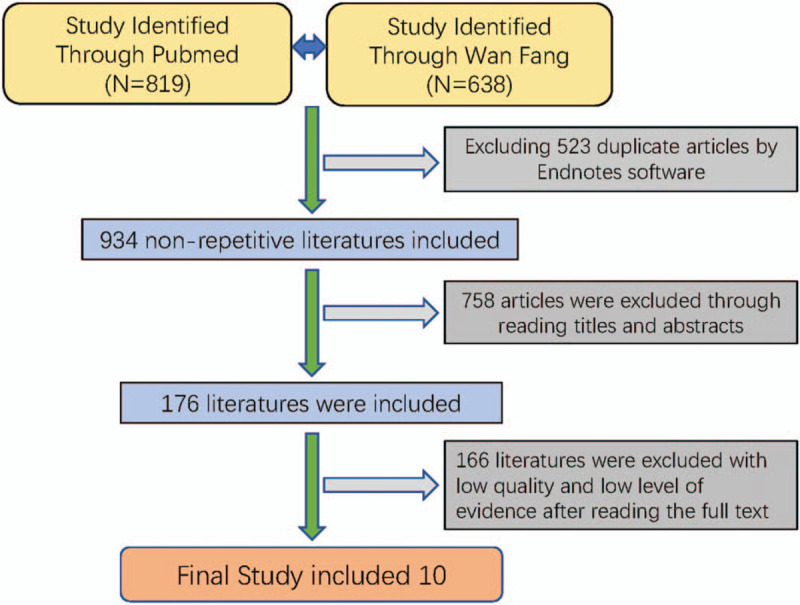
Flow chart of the study selection procedure.

Network meta-analysis was performed and relative data were searched by using the PubMed and WanFang databases. The publication date was set from 1974 to May 2019 and the language was restricted to English and Chinese. Through all fields of advanced, we used the following key words to search suitable literatures: “Coflex,” “decompression,” “PLIF,” “Posterior Lumbar Interbody Fusion,” “Coflex,” “Lumbar interbody Fusion.” Then we reviewed the titles and abstracts to select the potential articles. Finally, we carefully read the full texts and selected suitable articles according with the inclusion and exclusion criterion. As shown in Fig. 1.

### 2.2 Inclusion criteria

Literatures were included based on the following criteria: randomized controlled trials (RCTs); patients were diagnosed definite as lumbar degenerative disease; including clinical outcomes like Visual Analogue Scale (VAS) and Oswestry Disability Index (ODI) measurements, and complications; studies compared with 2 interventions including Coflex, decompression alone, and lumbar interbody fusion; complete data.

### 2.3 Exclusion criteria

The following studies should be excluded: case report; literature review; incomplete data; low quality.

### 2.4 Data extraction

Basic information, including study, design, interventions, age, sex, sample, and follow-up; clinical outcomes, including ODI, VAS; complications, including relapse, infection, dural sac rupture, venous thromboembolism (VTE), interventions loose, and no-union.

### 2.5 Quality assessment

Cochrane risk of bias tool was used in this study to conduct the quality assessment of RCTs.

### 2.6 Statistical analysis

Network meta-analysis with RCT model information was performed using a package gemtc in R 3.53. Continuous variables like ODI and VAS were analyzed using mean differences (MD) with its 95% credible interval (CrI). Then we performed a heterogeneity test to calculate the effects of direct and indirect comparisons. Furthermore, node-splitting analysis was made to estimate inconsistency by comparing the difference between direct and indirect effects. No significant inconsistency existed in outcomes if *P* value >.05.

## 3 Results

### 3.1 Identification

We retrieved 819 and 638 related studies through the PubMed and WanFan databases, respectively. Through endnotes software, we excluded duplicate articles. Then we read titles, abstract, and the full text, we excluded 1447 articles. A total of 10 eligible literatures were finally screened, including 946 patients (Table 1). All studies were RCT. VAS evaluations were included in all studies and only 1 study did not include ODI measurements. All included articles mentioned the complication of surgery.

**Table 1:**
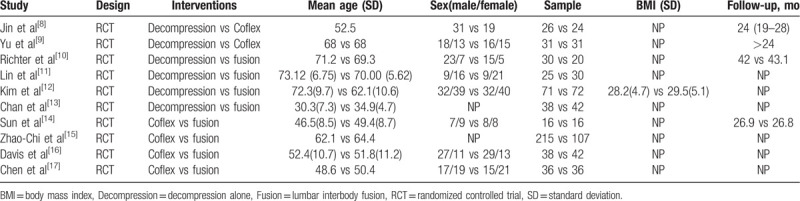
The characteristics of included studies.

### 3.2 Quality assessment

**Figure 2:**
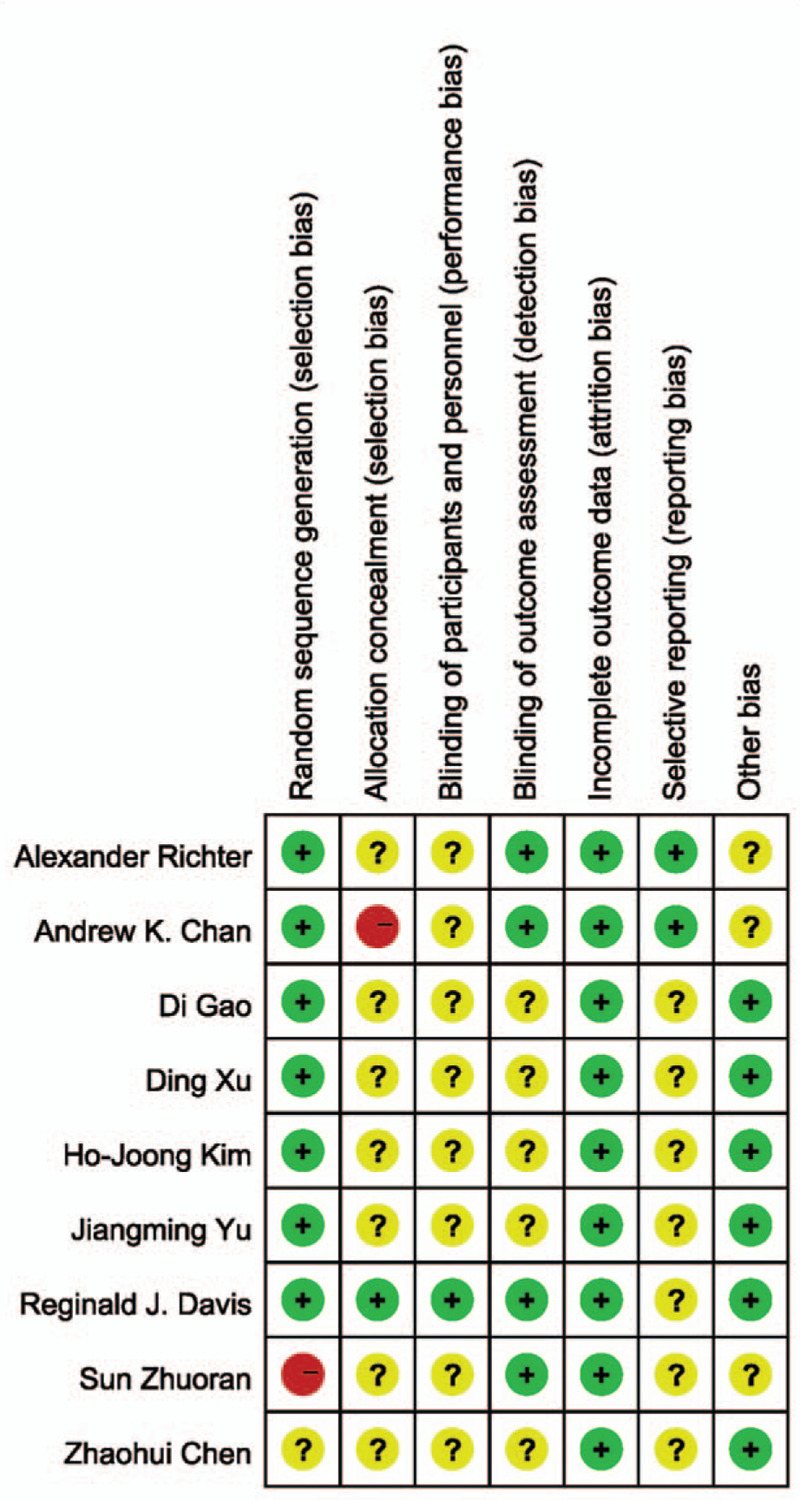
Risk of bias summary of RCT (Note: The yellow circle with question mark represents “unclear risk of bias,” the red one with minus sign represents “high risk of bias” and the green one with plus sign represents “low risk of bias”). RCT = randomized controlled trial.

**Figure 3:**
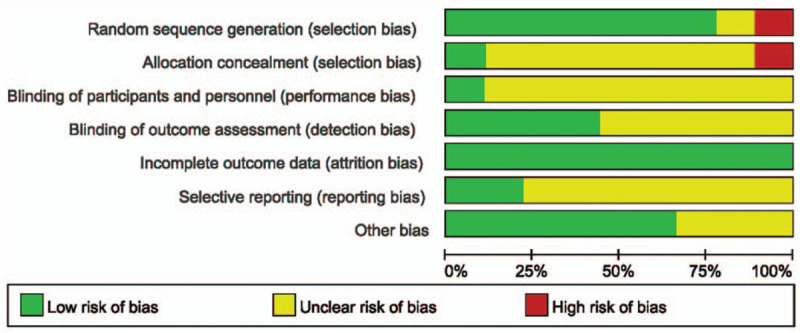
Risk of bias.

All 10 RCT studies were performed the quality assessment using the Cochrane risk of bias tool. The risk of bias summary is shown in Fig. 2 and the risk of bias figure was shown in Fig. 3. There was a high risk in Random sequence generation of Sun Zhuoran study and allocation concealment of Andrew K. Chan study.

### 3.3 The network results

**Figure 4:**
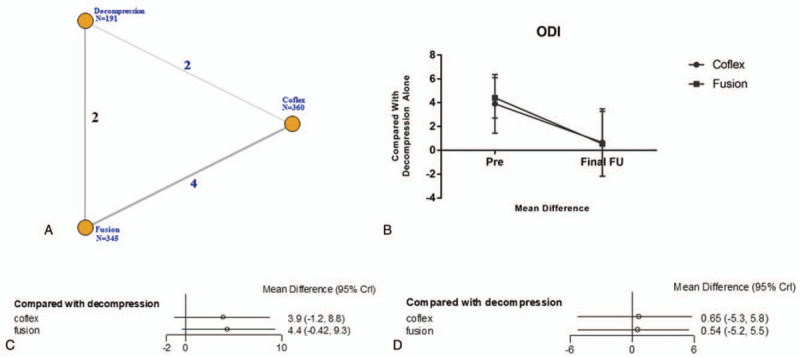
The clinical outcome of ODI. A: The network diagram. B: The trendline of ODI. C: The network forest of preoperation. D: The network forest of postoperation. ODI = Oswestry Disability Index.

Nine studies reported the ODI measurements and the data were completed (Table 2). Compared with decompression alone group, there was no significant difference of post-operation ODI score in Coflex and lumbar interbody fusion groups (Fig. 4). The figure showed that the preoperation MD of Coflex and fusion groups compared with decompression alone were 3.9 (95% CrI = –1.2, 8.8) and 4.4 (–0.42, 9.3), respectively. And the postoperation MD of Coflex and fusion were 0.65 (95% CrI = –5.3, 5.8) and 0.54 (95% CrI = –5.2, 5.5). The discrepancy between Coflex and fusion was not significant.

**Table 2:**
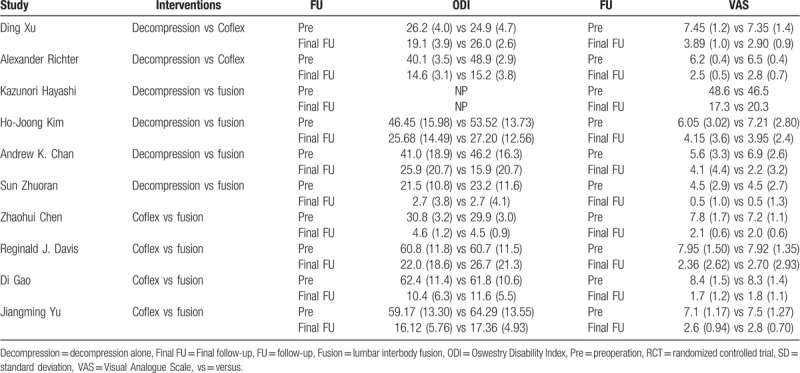
Clinical outcomes.

**Figure 5:**
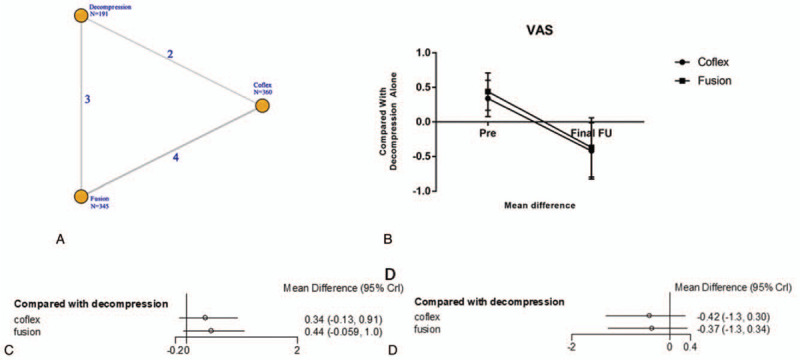
The clinical outcome of VAS. A: The network diagram. B: The trendline of VAS. C: The network forest of pre-operation D: The network forest of post-operation. VAS = Visual Analogue Scale

All studies reported VAS outcome (Table 2). Unlike ODI, we found that there was a significant difference in the postoperation VAS. Compared with lumbar interbody fusion, the postoperation MD of Coflex was –0.42 (95% CrI = –1.3, 0.30) and fusion was –0.37 (95% CrI = –1.3, 0.34). Before surgery, the MD in Coflex (MD = 0.34, 95% CrI = –0.13, 0.91) and fusion group (MD = 0.44, 95% CrI = –0.059, 1.0) was higher than decompression alone group. As shown in Fig. 5.

**Figure 6:**
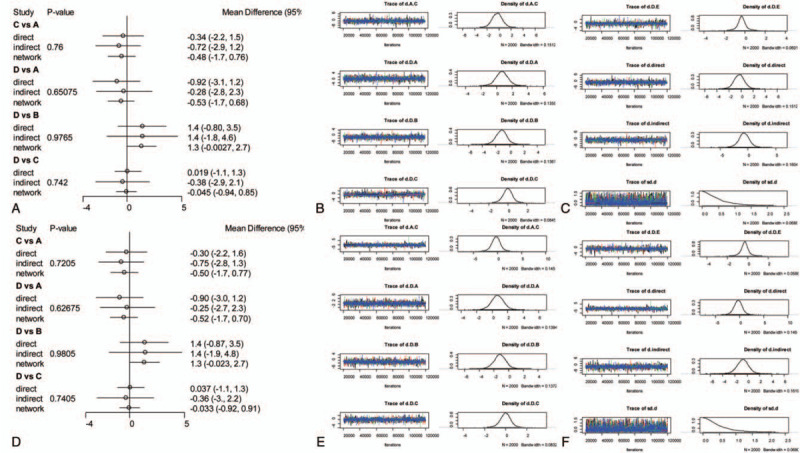
The network consistency chart of ODI. A: Preoperation consistency forest. B, C: Preoperation convergence graph. D: Postoperation consistency forest. E, F: Postoperation convergence graph. ODI = Oswestry Disability Index.

**Figure 7:**
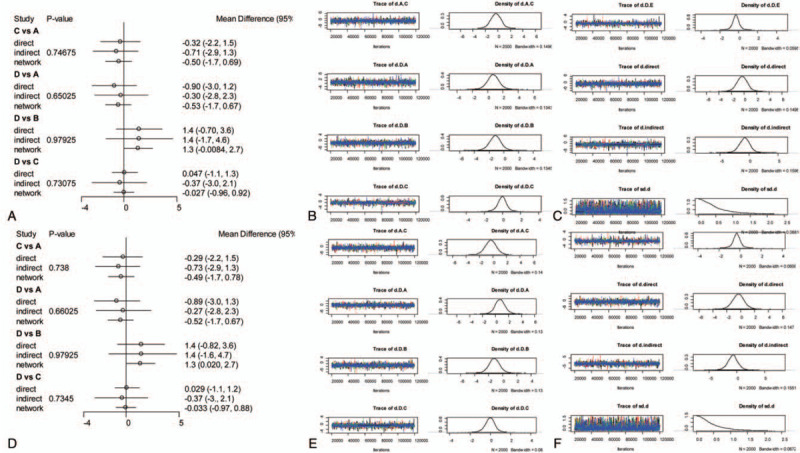
The network consistency chart of VAS. A: Preoperation consistency forest. B, C: Preoperation convergence graph. D: Postoperation consistency forest. E, F: Postoperation convergence graph. VAS = Visual Analogue Scale.

**Figure 8:**
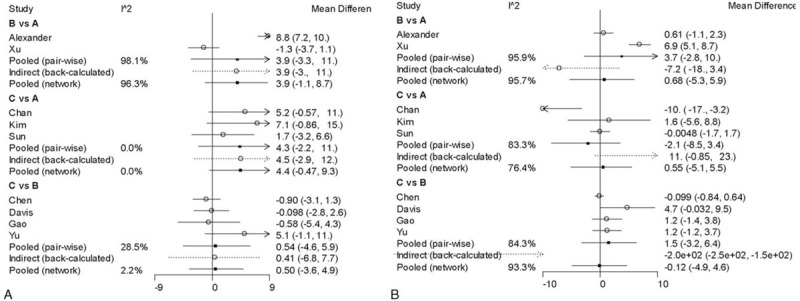
The heterogeneity chart of ODI. A: Preoperation heterogeneity forest. B: Postoperation heterogeneity forest. ODI = Oswestry Disability Index.

**Figure 9:**
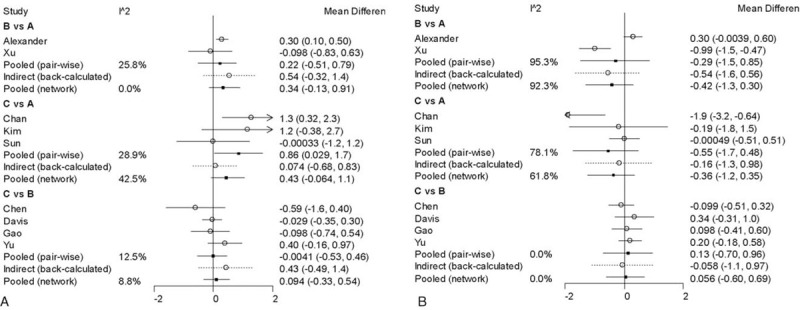
The heterogeneity chart of VAS. A: Preoperation heterogeneity forest. B: Postoperation heterogeneity forest.

A consistency chart (Figs. 6 and 7) and heterogeneity (Figs. 8 and 9) test were performed to reflect the degree of convergence of the model. We found no significant inconsistency or qualitative difference available in the outcomes. The analysis achieves good convergence efficiency.

### 3.4 Complication

There were 13 patients with decompression alone surgery complained adverse events including 8 relapse and 3 dural sac rupture. Four patients with Coflex technique were suffer from complications, including 2 dural sac rupture, 1 Coflex intervention loose, and 1 vertebral fracture. There were 14 patients with PLIF surgery occurred adverse events, including 3 relapse, 2 infection, 2 dural sac rupture, 1 VTE, 2 intervention loose, and 1 vertebral fracture as shown in Table 3.

**Table 3:**
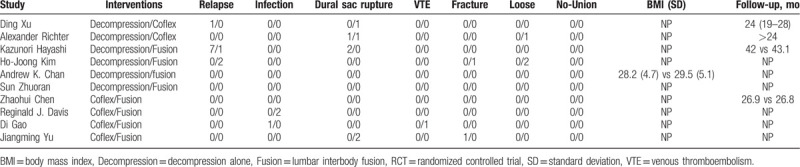
The complications of included studies.

## 4 Discussion

The comparative effect and safety of lumbar interbody fusion, Coflex, and decompression alone were not evaluated before. Decompression alone like minimally invasive surgery (MIS) relived pain and avoided further destabilization of spine, however it needed more technically demanding procedure and was associated with inferior outcomes.^[17–19]^ But Parker e al^[20]^ and Lu et al^[21]^ thought there were no significant differences between the 3 techniques regarding clinical outcomes and complications. To this end, we performed this network meta-analysis to compare the effectiveness and safety between decompression alone, interspinous device Coflex, and lumbar interbody fusion.

In the clinical outcome measurements, ODI was used to evaluate lumbar function and quality of life. And VAS was a useful indicator to evaluate patient's functional recovery. Although some articles also made a (Japanese Orthopaedic Association Scores [JOA]) assessment, the data we collected were not suitable to perform a network meta-analysis. All surgeries were aimed to relieve the compression of nerve caused by degenerative tissues or osteophyte and pain anesis.^[22,23]^

In this study, ODI score were declined significantly (*P* < .05) which proved all methods had improved the patient's lumbar function and quality of life. Patients with Coflex (MD = 3.9, 95% CrI = –1.2, 8.8) and lumbar interbody fusion (MD = 4.4, 95% CrI = 0.42, 9.3) general endured lower qualification of life compared with decompression alone. There was no a significant difference in ODI measurement and the postoperation MD of Coflex was 0.65 (95% CrI = –5.3, 5.8) and 0.54 in fusion group (95% CrI = –5.2, 5.5). The date indicated that decompression alone, Coflex, and lumbar interbody fusion had a similar effectiveness in improving the patients quality of life. Zhuomao et al^[24]^ also showed that no significant differences were found, however, decompression alone had a higher JOA score (*P* = .016) at the 3-month follow-up.^[25]^ The difference in improving quality of life was not significant in this study (MD 0.50, 95% CrI = –3.6, 4.9). And there was also no significant difference (*P* = .075 > .05) in Boden et al^[26]^ and Hambly et al^[27]^ studies.

In terms of VAS, this study showed that all techniques were useful to relive patient pain. Preoperation VAS was higher than decompression alone in Coflex (MD 0.34, 95% CrI = –0.13, 0.91) and fusion (MD 0.44, 95% CrI = –0.059, 1.0) group and that means the patients with Coflex and fusion techniques suffered from a more badly pain. Interestingly, the VAS of final follow-up in Coflex (MD –0.42, 95% CrI = –1.3, 0.30) and fusion (MD –0.37, 95% CrI = –1.3, 0.34) were decreased more significant. That is to say, the pain was relived more obviously compared with decompression alone after surgery. Some studies^[28–30]^ thought patients with Coflex and lumbar interbody fusion got an evident relief of pain, and the VAS of postoperation (Coflex 1.7 ± 1.2, fusion 1.8 ± 1.1) was decreased compared with preoperation (Coflex 8.4 ± 1.5, fusion 8.3 ± 1.4). Mardjetko et al^[31]^ showed that fusion group got a higher ΔVAS back pain score (–4.7 ± 3.2) than decompression alone (–1.5 ± 4.4) and *P* value <.001. Similar to our study, Moojen et al^[32]^ demonstrated both Coflex and fusion techniques would relieve patients pain and no difference was founded between these 2 surgeries (MD = 0.094, 95% CrI = –0.33, 0.54).

As for the prognosis, we summarized the complications of these 3 surgeries. Relapse happened in decompression 8 person and fusion group 2 persons. They were considered as inadequate decompression in the first surgery not as degenerative restenosis.^[33,34]^ There were 2 patients happened postoperation infection in decompression alone and fusion groups respectively.^[35]^ Persons with Coflex had a lower infection incidence rate. Interesting, all surgeries would result in dural sac rupture, so we suggested some attention should be paid to avoid this adverse event. We found the complication incidence rate of Coflex was lower than the others (*P* < .05).

## 5 Conclusions

Our network meta-analysis suggested that compared with decompression alone, Coflex and lumbar interbody fusion all can improve patients quality of life and relieve pain. The latter 2 techniques were better in relieving pain. Furthermore, Coflex performed a lower complication incidence rate compared with others. So we suggested that Coflex technique was a better choice to cue lumbar spinal stenosis (LSS).

## Acknowledgments

The authors thank Nanjing medical University library system for all of the help and resources and our deepest gratitude goes to the anonymous reviewers and editors for their careful work and thoughtful suggestions that have helped improve this paper substantially.

## Author contributions

YPF conceived and designed the study and wrote this manuscript. LLZ participated in interpretation of data, helped in drafting the manuscript and critically reviewed the manuscript. All authors read and approved the final manuscript.
